# Exogenous Ether Lipids Predominantly Target Mitochondria

**DOI:** 10.1371/journal.pone.0031342

**Published:** 2012-02-14

**Authors:** Lars Kuerschner, Doris Richter, Hans Kristian Hannibal-Bach, Anne Gaebler, Andrej Shevchenko, Christer S. Ejsing, Christoph Thiele

**Affiliations:** 1 Life and Medical Sciences Institute (LIMES), University of Bonn, Bonn, Germany; 2 Department of Biochemistry and Molecular Biology, University of Southern Denmark, Odense, Denmark; 3 Max Planck Institute of Molecular Cell Biology and Genetics, Dresden, Germany; Newcastle University, United Kingdom

## Abstract

Ether lipids are ubiquitous constituents of cellular membranes with no discrete cell biological function assigned yet. Using fluorescent polyene-ether lipids we analyzed their intracellular distribution in living cells by microscopy. Mitochondria and the endoplasmic reticulum accumulated high amounts of ether-phosphatidylcholine and ether-phosphatidylethanolamine. Both lipids were specifically labeled using the corresponding lyso-ether lipids, which we established as supreme precursors for lipid tagging. Polyfosine, a fluorescent analogue of the anti-neoplastic ether lipid edelfosine, accumulated to mitochondria and induced morphological changes and cellular apoptosis. These data indicate that edelfosine could exert its pro-apoptotic power by targeting and damaging mitochondria and thereby inducing cellular apoptosis. In general, this study implies an important role of mitochondria in ether lipid metabolism and intracellular ether lipid trafficking.

## Introduction

One fifth of all glycerophospholipids in humans are ether lipids featuring an alkyl chain at the *sn*-1 position and commonly an ethanolamine or choline head-group [1]. Highly abundant in certain tissues ether lipids are linked to pathologies and genetic disorders although their biological function remains unclear [2]. An involvement in membrane organization, fusion and trafficking or intracellular signaling and protection against oxidative stress is discussed [1], [3], [4]. Changed levels link to neurological dysfunction and degeneration [1], [5]. Inactivation of ether lipid biosynthesis in mice causes male infertility, defects in eye development, cataract and optic nerve hypoplasia [2]. Little detail of the subcellular localization of ether lipids is known; although synthesized in peroxisomes and the endoplasmic reticulum (ER), they also occur in the plasma membrane (PM), post-Golgi compartments and lipid droplets, LDs [1], [2], [6], [7]. Neutral ether lipids contribute 20% to the LD core and ether-phosphatidylcholine, ePC, and ether-phosphatidylethanolamine, ePE were detected in the surrounding monolayer [7].

Several synthetic ether lipids, the prototype being edelfosine (1-O-octadecyl-2-O-methyl-glycero-3-phosphocholine), show anti-neoplastic activity [8]–[10]. The metabolically stable edelfosine induces a selective apoptotic response in cancer cells, sparing normal cells [11], [12] unless these are in a proliferate state [13]. This selectivity arises from the internalization step [14]–[16] as the drug is only readily incorporating into cellular membranes of malignant cells [17], [18]. Edelfosine-induced apoptosis involves mitochondria [19], [20] and caspase-3 activation [20], but the underlying mechanism is complex as many cellular processes e.g. PC biosynthesis [21]–[23], signaling [24] and intracellular transport [25], [26] are affected.

Polyene lipids are fluorescent lipid analogues of high structural similarity to natural lipids [27]. Microscopy of polyene lipids holds the invaluable advantage of studying lipids in living cells avoiding compromises inherent to subcellular fractionation and purification approaches. Here, we employ polyene-ether lipids to visualize their subcellular distribution. Polyfosine, a fluorescent analogue of edelfosine, is used to elucidate the cellular localization of the bioactive compound and gain insights into its action.

## Materials and Methods

### Materials

Mitotracker Red CMXRos and Lysotracker Red DND-99 were from Invitrogen (Carlsbad, CA, USA). LD540 has been described [28]. Antibodies against Hsp60, Calnexin or cytochrome C were from Stressgen (Farmingdale, NY, USA), against active caspase-3 or COX IV from Cell Signaling (Danvers, MA, USA), against actin or tubulin from Sigma (Taufkirchen, GER), against Smac from MBL (Woburn, MA, USA).

**Figure 1:**
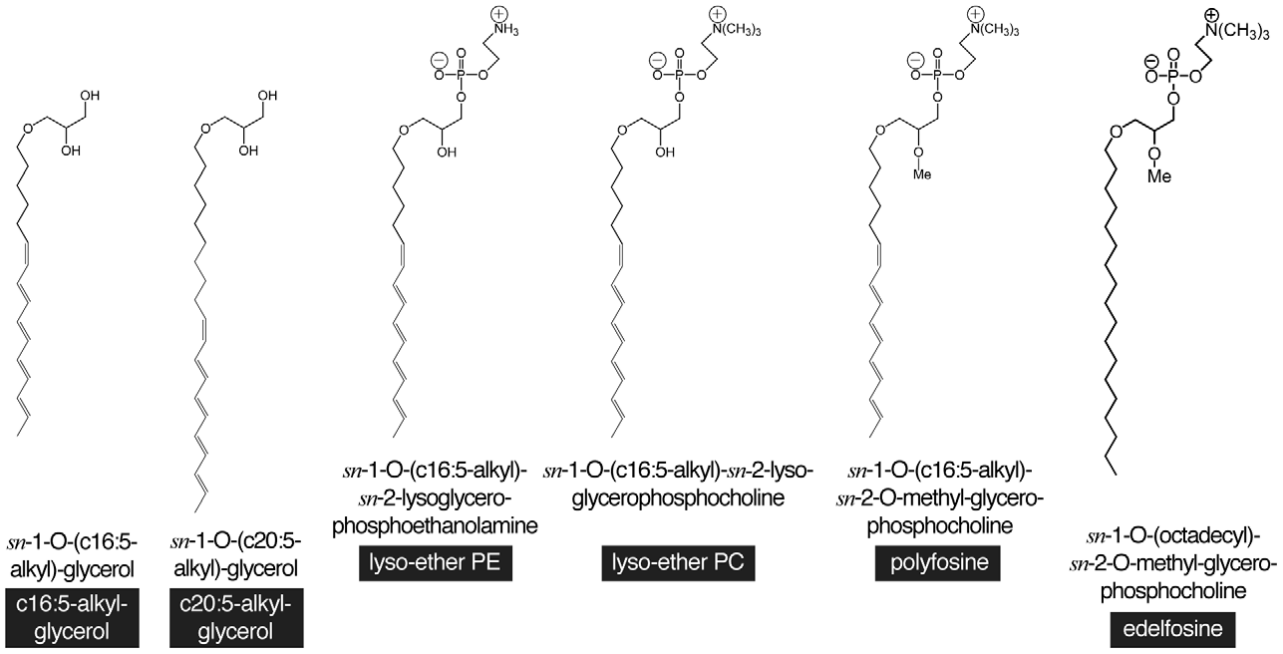
Ether lipids used in this study. Simplified names are black boxed. All lyso-lipids are *sn*-2-lyso-lipids.

Edelfosine was from Sigma, other ether lipids (Fig. 1) were synthesized as described in Text S1.

### Methods

COS7 (from ATCC®, Number: CRL-1651) cell culture and delivery, extraction, TLC and detection of lipids were performed as described [27].

Quantification of signals on TLC plates and Western blotting films was performed using ImageGaugeV3.3 (Fuji, Duesseldorf, GER).

### Mass spectrometry (MS)

Cells were washed sequentially with PBS, 155 mM ammoniumacetate pH6.9 and HES (2 mM Hepes, 1 mM EDTA, 250 mM sucrose, pH 7.0) and scraped. Lipids were extracted as described [29]. Lipids were dissolved in chloroform/methanol/2-propanol (1∶2∶4) containing 7.5 mM ammoniumacetate and analyzed by multiple precursor ion scanning on a QSTAR-Pulsar-i quadrupole time-of-flight mass spectrometer (AB SCIEX, Concord, ON, CAN) equipped with a robotic nanoflow ion source Triversa NanoMate (Advion Biosciences Inc., Ithaca, NJ USA) [30], [31]. Plasmanyl-PC species were detected by precursor ion scanning (PIS) *m/z* 456.3 analysis [32]. Plasmanyl-PE and plasmenyl-PE species were detected by PIS *m/z* 428.2 and PIS *m/z* 426.2 analyses, respectively [33]. Detected lipid precursors were identified using Lipid View software (AB SCIEX) [31]. Alternatively, endogenous PC, ePC, PE and ePC species were profiled by high-resolution positive ion mode fourier transform MS analysis on a LTQ Orbitrap XL mass spectrometer (Thermo Fisher Scientific) equipped with a Triversa NanoMate.

### Cell fractionation

Cells were washed and collected as above, followed by homogenization in a cooled EMBL cell cracker (HGM, Heidelberg, GER) with 8 strokes using a maximum clearance of 18 µm. To purify the mitochondria by differential centrifugation the supernatant (PNS, post nuclear supernatant) after a first centrifugation step (100–1,000×g; 2–5 min, respectively) was separated from the pellet (PNP, post nuclear pellet) and centrifuged again (10,000×g; 10 min) to obtain a crude mitochondria pellet (PMP, post mitochondrial pellet) and a supernatant (PMS, post mitochondrial supernatant). The PMP was redissolved in HES and layered on top of a step gradient (40%, 26% and 12% Percoll in HES). The gradient was centrifuged (25 min, 150,000×g) before harvesting 8 fractions from the top. Samples were subjected to SDS-PAGE and Western blotting.

### Microscopy

Two-photon-excited fluorescence microscopy of living cells was performed as described [27]. Epifluorescence microscopy of living and fixed cells was performed using a Zeiss Observer.Z1 microscope (Carl Zeiss, Oberkochen, GER) equipped with a C-Apochromat 63× (1.20 NA) and a Photometrics Evolve camera, or a Plan-Apochromat 63× (1.40 NA) DIC and a Photometrics Coolsnap K4 camera, respectively. Live cells imaging was performed at 37 degree C in a 5% carbondioxid atmosphere. Light source was a Polychrome V 150 W xenon lamp (TillPhotonics, Gräfelfing, GER). Confocal immuno-fluorescence laser scanning microscopy of fixed cells was performed using a Leica TCS SP2 microscope (Leica, Wetzlar, GER) equipped with a HCX PL APO CS 40× (1.25 NA). Relief contrast bright field microscopy of living cells was performed using an Olympus CKX31 microscope (Olympus, Hamburg, GER) equipped with a LCACHN 20xRC (0.4 NA) and a halogen light source. Images were acquired with Canon Powershot digital camera (Canon, Amsterdam, NED). Phase-contrast video microscopy of living cells was performed using an Olympus IX70 microscope (Olympus) equipped with a U-Plan S Apo 100× (1.40 NA) and a primary halogen lamp light source. A secondary light source, a Polychrome II 75 W xenon lamp (TillPhotonics) with its soft shutter wavelength set to 280 nm, and an FT395-LP415 (Carl Zeiss) was used to illuminate the cells continuously with broadband UV light. Digital images were acquired with a NTE/CCD-512-EBFT camera (Roper Scientific, Ottobrunn, GER). All images were processed employing Adobe Photoshop 6.0 (Adobe).

## Results

### Polyene-ether lipids derived from alkyl glycerol precursors

**Figure 2:**
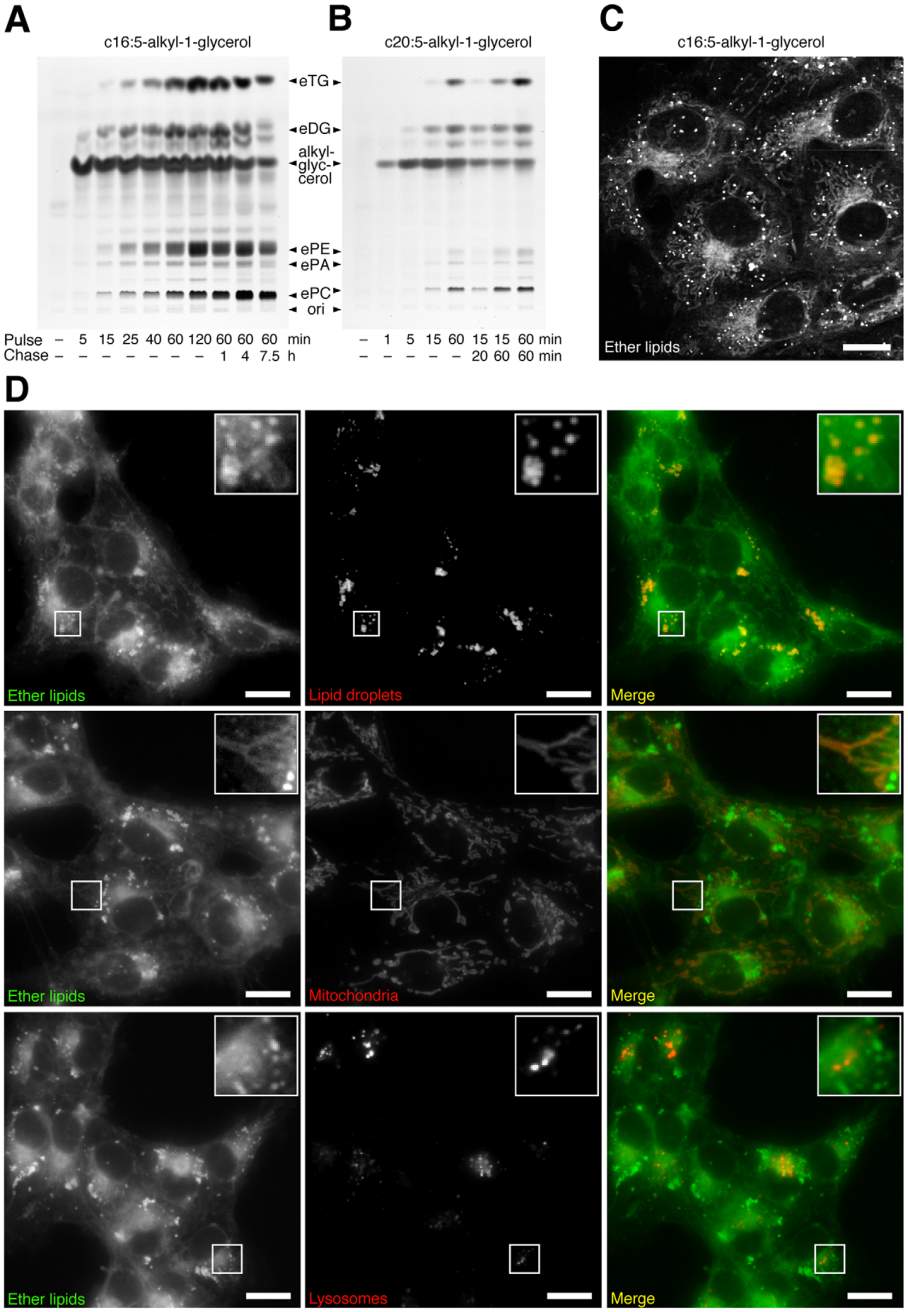
Metabolism and intracellular distribution of polyene-alkyl-glycerols in COS7 cells. Cells were incubated with 50 µM of c16:5-alkyl-glycerol (A, C, D) or c20:5-alkyl-glycerol (B) for different pulse times (A & B), 2 h (C) or 0.5 h (D). Cellular lipids were extracted and analyzed by TLC for fluorescent metabolites, which were identified by comigrating lipid standards (A & B). Living cells were imaged using two-photon-excitation microscopy (C) or epifluorescence microscopy (D). Merged color images show ether lipids in green and LDs, mitochondria, or lysosomes stained by LD540, Mitotracker, or Lysotracker, respectively, in red (D). Bars, 20 µm. *ori*, origin of application.

We fed polyene-alkyl glycerols to cells and analyzed the fluorescent metabolic products by TLC. From 50 µM concentrations of c16:5-alkyl-glycerol or c20:5-alkyl-glycerol, the cells produced primarily neutral ether lipids (ether-diglycerides, eDG; ether-triglycerides, eTG) and ether glycerophospholipids (ePE, ePC and ether-phosphatidic acid, ePA) during the experiment (Fig. 2A and B). Differences in alkyl chain lengths did not influence the labeling pattern, also when 3 µM concentrations were used (data not shown). Considerable amounts of precursor, whose concentration decreased only slowly during a chase, were detectable. Studying the distribution of polyene-ether lipids in living cells by two-photon-microscopy (Fig. 2C) revealed a staining of the nuclear envelope, ER, LDs and mitochondria. To unequivocally identify the latter we performed epifluorescence microscopy colocalization studies (Fig. 2D). LDs and mitochondria were confirmed to contain polyene-ether lipids. Peroxisomes did not accumulate ether lipids as shown before [27].

### Polyene-ether lipids derived from lyso-ether lipid precursors

**Figure 3:**
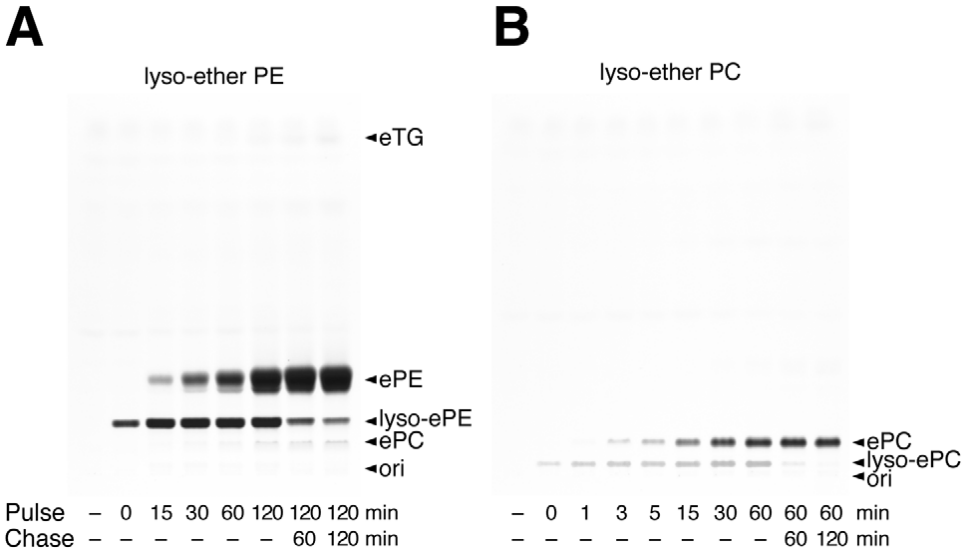
Metabolism of polyene-lyso-etherlipids in COS7 cells. Cells were incubated with 50 µM of c16:5-lyso-ePE (A) or c16:5-lyso-ePC (B) for different pulse times. Fresh medium was applied for chase times. Lipid were extracted and analyzed by TLC for fluorescent metabolites, which were identified by comigrating lipid standards.

**Figure 4:**
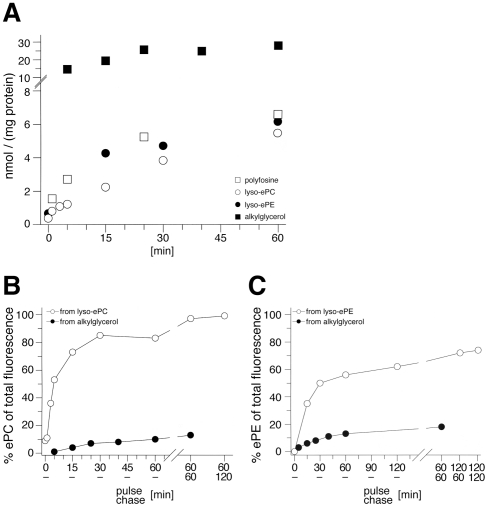
Uptake and labeling specificity of various polyene-ether lipid precursors. The total amount of fluorescent lipids in COS7 was determined by fluorescence spectroscopy and plotted over incubation time (A). Fluorescent ePC (B) or ePE (C), derived from c16:5-alkyl-1-glycerol (B & C, closed circles) or c16:5-lyso-ePC (B, open circles) or c16:5-lyso-ePE (C, open circles), respectively, was quantified from TLC plates (Figs. 2 & 3). Results from a single experiment are displayed as background-corrected percent values of total fluorescence in the TLC lane.

A shortcoming of using alkyl glycerols for ether lipid tagging is the limited specificity. Labeling selectivity was greatly increased when cells were incubated with polyene lyso-ether lipids. Application of polyene-lyso-ePE yielded fluorescent ePE upon cellular acylation (Fig. 3A). At later timepoints (60–120 min) minimal labeling of ePC could be detected as also natural ePC is synthesized from ePE [1]. During a 2 h chase the precursor concentration was reduced by 70%. When cells were incubated with polyene-lyso-ePC, cellular acylation yielded ePC with high specificity and rate (Fig. 3B). During the 2 h chase the precursor was converted to only ePC. Uptake and specificity of various polyene precursors were quantified (Fig. 4). The alkyl glycerol was taken up 5-fold more efficiently (Fig. 4A), but after 60 min incubation the lyso-ether lipids showed a 8-fold or 5-fold higher labeling specificity for the desired lipid class (Fig. 4B and C), which increased further during a chase.

To identify the fatty acids used for cellular acylation of polyene lyso-ether lipids MS analyses were performed (Table 1). Arachidonic acid (20:4) was the predominant acylation partner for both polyene-lyso-ePE and -ePC. Other endogenous unsaturated fatty acids, oleate (18:1), linoleate (18:2), and palmitoleate (16:1) were also detected. These findings are expected, since ePE and ePC species are rich in polyunsaturated fatty acids in untreated COS7 cells (Fig. S1) and other cells [1]. The 6-fold higher abundance of palmitate (16:0) and the presence of myristate (14:0) in ePC, but not in ePE (Table 1) might point to a different specificity of the acyltransferases with a higher acceptance of saturated acyl chains in ePC compared to ePE. The concentration of vinylated polyene plasmenyl-PE was 24-fold lower than that of polyene plasmanyl-PE. Due to peak overlay plasmenyl-PC could not be analyzed. MS analysis did not indicate any significant conversion of polyene ePE or ePC into ether-phosphatidylserine, ePS, or ePA. Taken together, polyene-lyso-ether lipids are acylated by cellular enzymes without apparent disturbances by the tag.

**Table 1:** MS analysis of ether glycerophospholipids from COS7 cells incubated with 50 µM polyene-lyso-ePE or -ePC for 2 or 1 h, respectively.

label	*m/z*	lipid species	rel. int. [%]	*m/z*	lipid species	rel. int. [%]
lyso-	638.4	ePE(O-16:5/14:0)	2	636.4	ePE(P-16:5/14:0)	2
ether-	664.4	ePE(O-16:5/16:1)	23	662.4	ePE(P-16:5/16:1)	17
PE	666.5	ePE(O-16:5/16:0)	8	664.4	ePE(P-16:5/16:0)	10
	690.5	ePE(O-16:5/18:2)	21	688.4	ePE(P-16:5/18:2)	25
	692.5	ePE(O-16:5/18:1)	45	690.5	ePE(P-16:5/18:1)	45
	694.5	ePE(O-16:5/18:0)	2	692.5	ePE(P-16:5/18:0)	8
	714.5	ePE(O-16:5/20:4)	100	712.4	ePE(P-16:5/20:4)	100
	716.5	ePE(O-16:5/20:3)	8	714.5	ePE(P-16:5/20:3)	13
lyso-	740.5	ePC(O-16:5/14:0)	22			
ether-	766.5	ePC(O-16:5/16:1)	36			
PC	768.5	ePC(O-16:5/16:0)	55			
	792.5	ePC(O-16:5/18:2)	40			
	794.5	ePC(O-16:5/18:1)	21			
	796.5	ePC(O-16:5/18:0)	3			
	814.5	ePC(O-16:5/20:5)	14			
	816.5	ePC(O-16:5/20:4)	100			
	818.5	ePC(O-16:5/20:3)	15			

“O” indicates plasmanyl, “P” plasmenyl lipids.

### Ether lipids in mitochondria and ER

**Figure 5:**
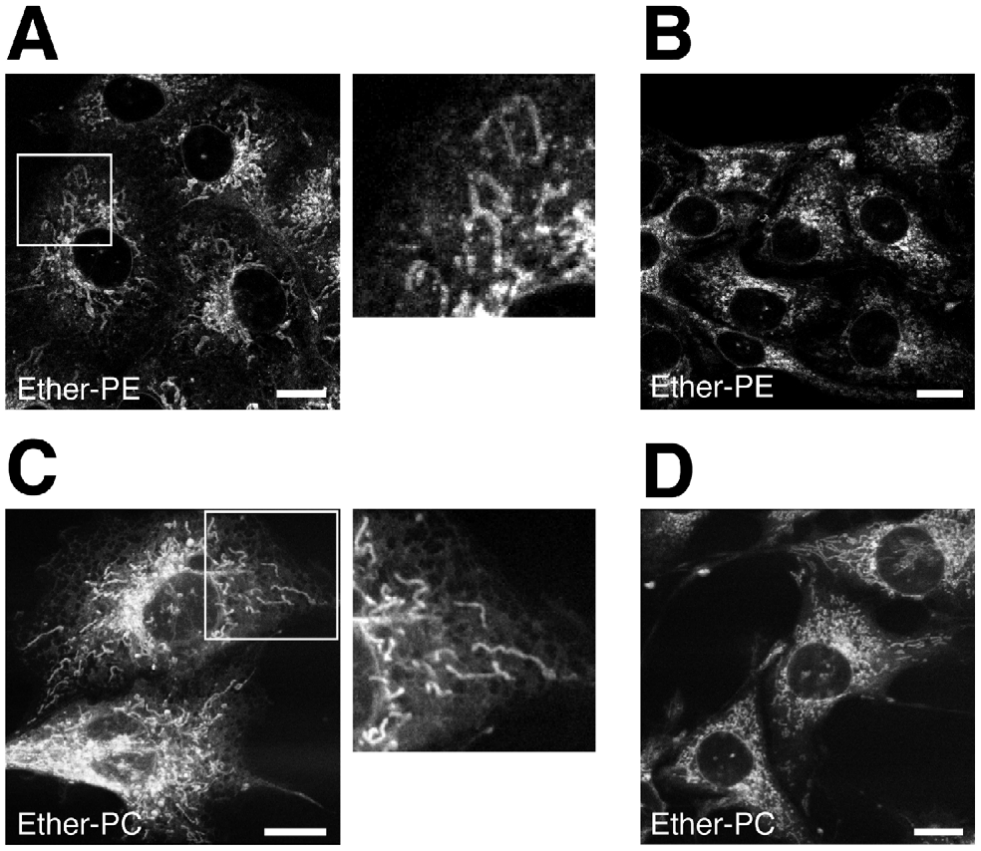
Imaging of polyene-ether phospholipids in COS7 cells. Cells were incubated with 50 µM lyso-ePE (A & B) or lyso-ePC (C & D) for 1 h (A & C) or 1 h+2 h chase (B & D). Fresh medium was applied for chase times. Cellular metabolism generated mostly ePE (A & B) or ePC (C & D) (see Fig. 3 A & B, respectively). Living cells were imaged using two-photon-excitation microscopy. Note, that the reticular ER staining appears less defined after the chase (B & D). Bars, 20 µm.

The specific labeling of ePE or ePC obtained using the respective polyene-lyso-ether lipid allowed for the localization of fluorescent ePE and ePC on the subcellular level in living cells for the first time. Two-photon-excitation microscopy revealed that ePE and ePC prominently stained the mitochondria, nuclear envelope and ER (Fig. 5). LD staining was not detected, as only minor amounts of fluorescent neutral ether lipids were biosynthesized during the experiment (Fig. 3).

**Figure 6:**
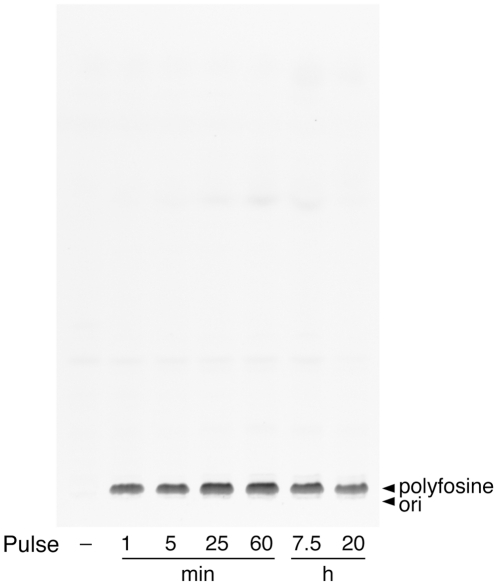
Metabolism of polyfosine in COS7 cells. Cells were incubated with 50 µM of polyfosine for different times. Lipids were extracted and analyzed by TLC for fluorescent metabolites, which were identified by comigrating lipid standards.

**Figure 7:**
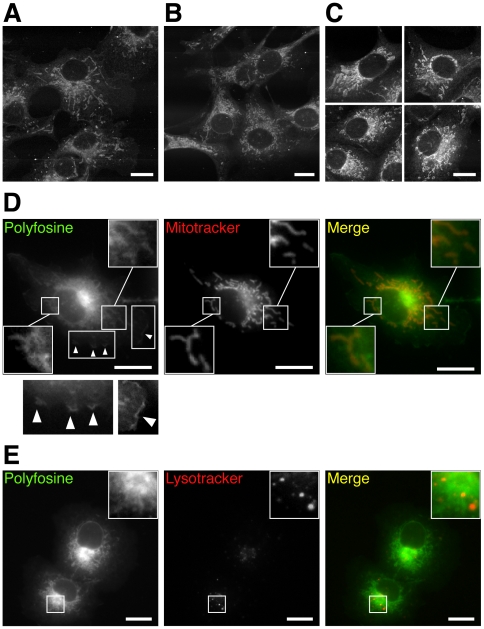
Imaging of polyfosine in COS7 cells. Cells incubated with 50 µM of polyfosine for 1 h (A, D & E), 2.5 h (B), or 5 h (C) were imaged by two-photon-excited lipid fluorescence (A–C) or epifluorescence (D & E) microscopy. Merged color images show polyfosine in green (D & E) and mitochondria (D) or lysosomes (E) stained by Mitotracker or Lysotracker, respectively, in red. Polyfosine stained convoluted PM ruffles (D, arrow heads). Bars, 20 µm.

When cells were incubated with polyfosine, a fluorescent analogue of the metabolically stable, synthetic ether lipid edelfosine, no significant metabolites could be detected within 20 h (Fig. 6). Fluorescence microscopy analysis revealed a pronounced polyfosine staining of mitochondria with a noticeable marking of nuclear envelope, ER and PM (Fig. 7). Long incubation with polyfosine resulted in shortened or swollen mitochondria (Fig. 7B and C). Colocalization studies confirmed the accumulation to mitochondria (Fig. 7D), while lysosomes contained hardly any detectable polyfosine (Fig. 7E).

### The impact of polyfosine and edelfosine on mitochondria

**Figure 8:**
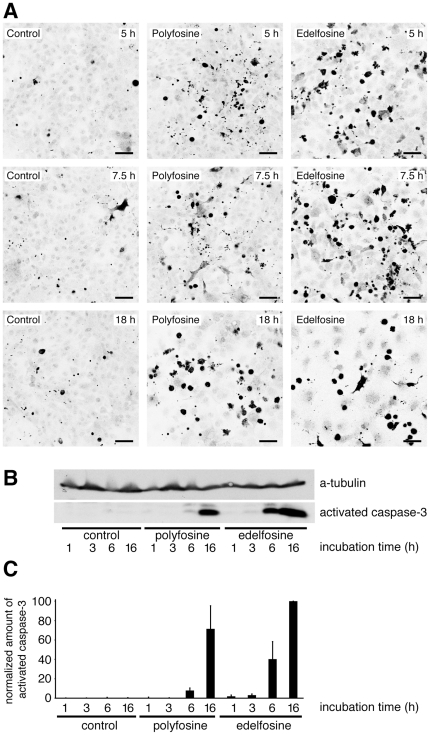
Induction of apoptosis in COS7 cells by polyfosine or edelfosine. (A) Cells incubated with 10 µM of polyfosine or edelfosine for indicated times were fixed and probed for activated caspase-3, a landmark of apoptosis. After 18 h many apoptotic cells had detached from support. Confocal laser immuno-fluorescent microscopy images are shown as inverted grayscale. Bars, 50 µm. (B & C) Lysates of cells incubated with 10 µM of polyfosine or edelfosine for indicated times were assayed by Western blotting for activated caspase-3 and for a-tubulin, which served as a load control. (C) Signal intensities from four Western blots were quantified. Shown is the amount of activated caspase-3 corrected for background and normalized to the a-tubulin signal. The amount of active caspase-3 in edelfosine treated cells was set to 100.

To compare the potentials of edelfosine and polyfosine to induce cellular apoptosis the activation of caspase-3 in immortal COS7 cells was monitored by microscopy and Western Blotting (Fig. 8). The number of caspase-3 positive cells and its concentration increased over time, faster for edelfosine compared to polyfosine treatment. Long incubation times resulted in decreased cell viability and number, due to apoptosis-induced detachment (Fig. 8A and Fig. S2).

**Figure 9:**
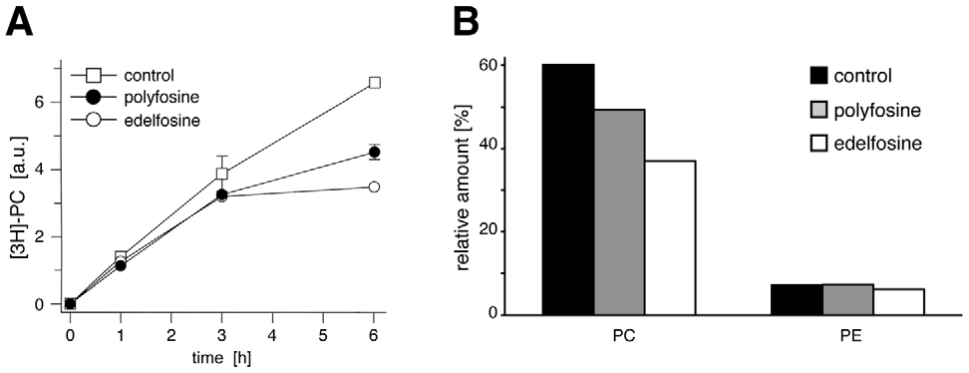
Inhibition of PC synthesis. (A) COS7 cells were incubated with 10 µM polyfosine (closed circles) or edelfosine (open circles) in the presence of [3H]-myristic acid. At indicated times lipids were extracted and analyzed by TLC for radioactive metabolites. The signal intensity of labeled PC was quantified from TLC plates. Data are mean values ± range, n = 2. Error bars smaller than symbol size are omitted. (B) COS7 cells were incubated with 50 µM polyfosine or edelfosine for 1 h. Cellular lipids were extracted and analyzed by MS. The total PC and PE intensity was normalized to the sum of all major lipids (PC, PE, SM, DG, TG, ePC and ePE). Data are mean values, n = 2, with a range of less then three percent.

To compare the inhibition of PC biosynthesis by edelfosine and polyfosine the incorporation of a radio-labeled fatty acid into PC was assayed (Fig. 9A). After an initial decrease in the incorporation rate, edelfosine treated cells stopped the synthesis and remodeling of PC. Longer polyfosine treatment also reduced the amount of labeled PC, although less efficiently. To corroborate these findings we analyzed the major cellular lipid classes by MS (Fig. 9B). The PC and PE contents were quantified relative to the sum of major lipids. After 1 h treatment, edelfosine decreased the total PC content by about 38%, polyfosine by 18% compared to control cells (Fig. 9B).

The morphological changes of mitochondria upon polyfosine accumulation were studied by fluorescence microscopy employing a dye sensitive to mitochondrial activity and vitality (Fig. S3A). In control cells mitochondria were often elongated and stained with similar intensities. Many polyfosine and all edelfosine treated cells had short and fragmented mitochondria (Fig. S3B). While many apoptotic cells upon polyfosine treatment did not accumulate the dye, edelfosine treated cells with condensing nuclei or arrested in mitosis often showed intensively stained mitochondria with an increased cellular background stain (Fig. S3A). Mitochondrial fragmentation leads to changes in organelle size, shape and density, which prevented their isolation by density gradient centrifugation to a satisfying purity from contaminating ER (Fig. S4).

**Figure 10:**
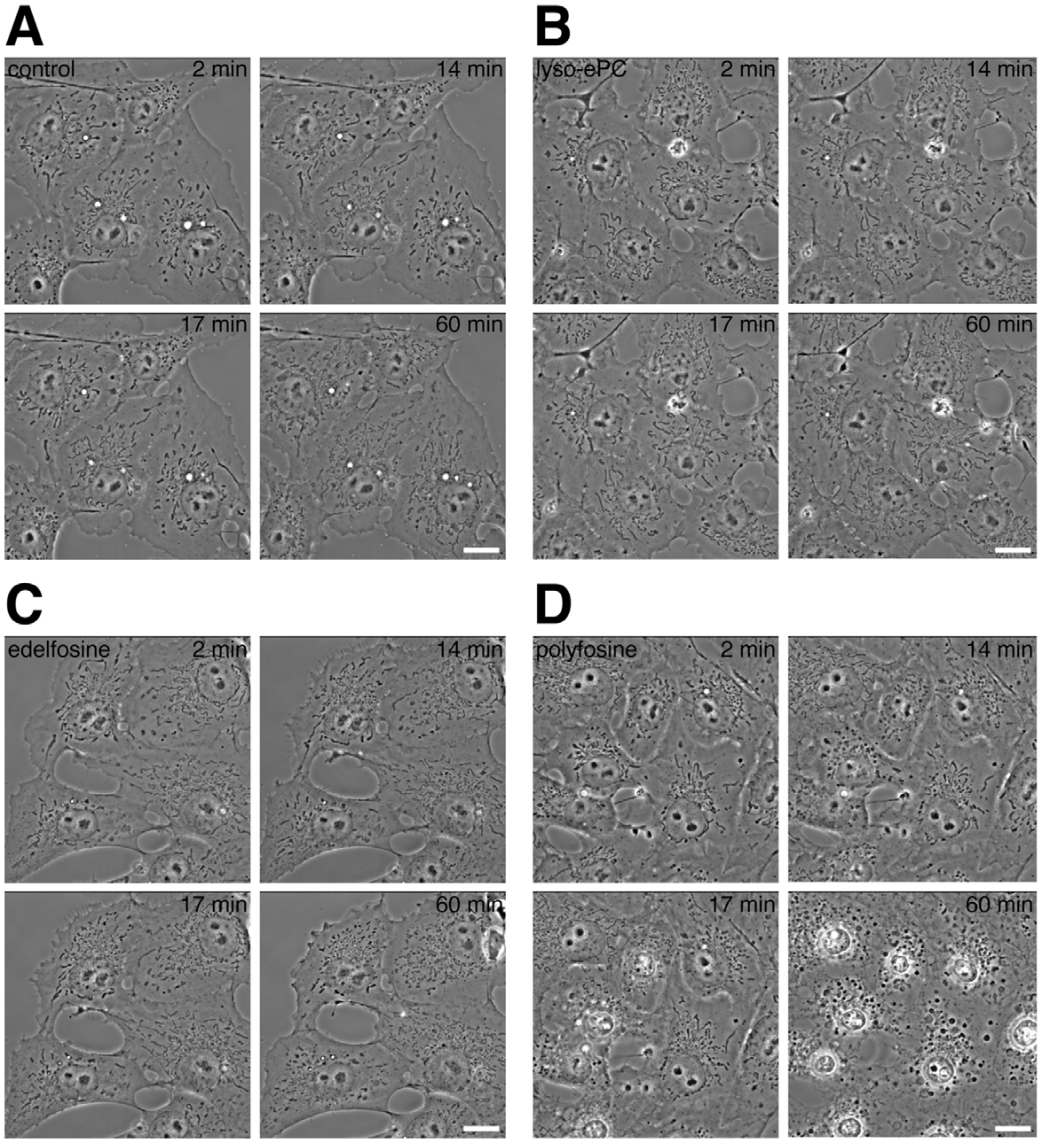
UV light induced phototoxicity. COS7 cells were incubated with carrier (A) or 50 µM of lyso-ether PC (B), edelfosine (C) or polyfosine (D). Living cells were imaged by phase-contrast video microscopy for at least 60 min, during which a broadband UV illumination from a second light source was constantly applied. Bars, 20 µm. Mitochondria fragmented (C & D) and disintegrated (D). See also Movie S1.

Mitochondrial fragmentation was analyzed by phase-contrast video microscopy (Fig. 10 and Movie S1). After 10 min some polyfosine and edelfosine treated cells started mitochondrial fragmentation, while others followed after 15–25 min. Shortly after fragmentation the mitochondria of polyfosine, but not edelfosine treated cells condensed and disintegrated, followed by nuclear condensation. Control cells incubated with carrier or polyene-lyso-ePC, the latter sharing the fluorophore and the cellular localization with polyfosine (Fig. 5C and D) showed no morphological changes. Blebbed mitochondria upon polyfosine treatment were also observed in experiments evaluating an end-point (Fig. S5) but disintegration was significantly accelerated during video microscopy likely caused by the continuous UV illumination of the imaged cells. Apparently, the phototoxicity potentiated the membrane-disrupting effects of fluorescent polyfosine, but not edelfosine, known for its radiosensitizing properties [34].

## Discussion

Information on the subcellular localization of most ether lipids is limited. Using polyene lyso-ether lipids, which upon cellular acylation yielded fluorescent ePC or ePE, allowed for their unprecedented individual microscopic analysis. Both ether phospholipids strongly accumulated to mitochondria, besides the ER. The fact that fluorescent ePC or ePE was not detectable on LD monolayers [7] or the PM [4] likely reflects limitations in optical resolution and detection sensitivity. Polyene-sphingolipids were not accumulating on mitochondria arguing against an erratic targeting of the fluorophore to mitochondria [27]. Comparing the labeling patterns of polyene lyso-ether lipids and alkylglycerols we established lyso-ether lipids as supreme precursors for phosphoether lipid tagging as they represent a good compromise between labeling specificity, similarity to natural lipids and applicability to cells. Cell derived polyene-ePC and -ePE, like natural ether glycerophospholipids [1], often contained unsaturated fatty acids most prominently arachidonic acid. Placed to mitochondria ether lipids might serve as a sink for harmful oxidants resulting from oxidative phosphorylation. However, only a small amount of polyene-ePE was detected as vinylated plasmenyl-ePE with antioxidative properties [2]. Alternatively, mitochondria undergo extensive fusion and fission cycles [35] as do synaptic vesicles, which show high plasmalogen concentrations [36]; ether phospholipids might facilitate membrane reorganization in both cases.

After lyso-ether lipid feeding mitochondria became the first labeled organelles justifying speculations of the underlying targeting mechanism. The structure of lyso-ether lipids supports a non-facilitated transport between membranes. For instance, the related lyso-PC is spontaneously released from membranes within milliseconds, which allows for rapid kinetic equilibration [37]. Its release and cytosolic transport are supported by fatty acid binding proteins [38]. Mitochondria may provide the enzyme for lyso-ether lipid acylation, which would arrest the resulting ether lipid in the mitochondrial membrane. Tafazzin, a phospholipid acyltransferase catalyzing transacylation between lyso-PC and cardiolipin [39] represents a candidate.

Alternatively, ePC and ePE generated by non-mitochondrial acyltransferase(s) could be targeted to mitochondria by a mechanism also accessible to the synthetic ether lipids polyfosine and edelfosine. Especially for the latter, a raft-dependent endocytosis step has been discussed and is supported by the enrichment of both edelfosine [16], [40] and ether lipids [4] in rafts. Alternatively, membrane continuities between organelles could be involved as reported for lipid trafficking before [41]. Noteworthy, many other cellular organelles including LDs, which act as a cellular sink for hydrophobic xenobiotics [42] did not accumulate polyfosine, arguing for a specific rather than a stochastic enrichment at mitochondria.

One may not expect polyfosine enrichment in mitochondria as edelfosines established molecular targets are located to the ER and PM [16]. Labeling by a fluorescent phenylpolyene-analogue of edelfosine was restricted to the PM in leukemic cells or predominantly found in the ER of solid carcinoma yielding a model where the cell specific distribution pattern correlates with different cellular targets [43], [44]. A recent study performed in HeLa cells uses fluorescent borondifluorodipyrromethene (BODIPY) coupled edelfosine analogues and confocal laser microscopy to describe a mitochondrial localization of these probes [45]. Polyfosine, the analogue matching best the chemical structure of edelfosine confirms mitochondria as a target for the drug in COS7 cells, while verifying an ER and PM localization. Cell type or different cellular handling of the various analogues may explain the discrepancy in localization of the fluorescent analogues. Mitochondrial localization may be hard to detect if only non-confocal microscopic images are available [43], [44] as ER staining can interfere.

While polyfosine and edelfosine activated caspase-3 and interfered with PC biosynthesis our study supports the notion that the accumulation of these lipids to mitochondria per se results in a structural damage of the organelle that ultimately leads to apoptosis. These inverted cone-shaped lipids possibly generate membrane curvature if high local concentrations are met [46]. Indeed, edelfosine induced swelling of isolated mitochondria [45] and membrane destabilization in liposome model systems by formation of interdigitated structures, micelles and small vesicles [47]. Edelfosine's detergent properties [10] and very low critical micellar concentration were found to cause lipid solubilization and, importantly, release of content from lipid vesicles [48]. Physical membrane destabilization and loss of mitochondrial integrity results in release of mitochondrial proteins like cytochrome c [49], which ultimately induces apoptosis [35].

In summary, this study establishes polyene lyso-ether lipids as supreme precursors for phosphoether lipid tagging. Fluorescent phosphoether lipids accumulated to mitochondria, suggesting an important role for the organelle in lipid trafficking.

## Supporting Information

Figure S1 **Analysis of glycerophosphatidylcholine and glycerophosphatidylethanolamine lipids species of COS7 cells by MS.** Cells were grown in DMEM containing 4.5 g/ml glucose, GlutaMax I, pyruvate and 10% FCS. Total lipid extracts of cells were analyzed for the PC and ePC (A), or PE and ePE (B) species by high-resolution FT MS analysis. The intensity of each species was quantified as a fraction of the sum of all glycerophosphatidylcholine (A) or glycerophosphatidylethanolamine (B) species monitored. Lipid species with a relative amount of less than one percent are omitted.
(TIF)

Figure S2 **Analysis of the viability of COS7 cells upon treatment with polyfosine or edelfosine.** Cells were incubated with 10 µM of polyfosine, edelfosine or carrier as control for indicated times. Representative relief contrast microscopy images of the morphological appearance of the cells are shown (A). Note, that after 3–6 h of incubation with either polyfosine or edelfosine cells were rounding up and detaching from the support presumably by apoptosis-induced detachment. The nuclei appeared granular and fragmented. The number of apoptotic cells as judged by their morphology (detached or rounded up cells with granular fragmented nuclei) were counted and used to calculate the percentage of viable cells (B).
(TIF)

Figure S3 **Morphological changes of mitochondria and nuclei upon polyfosine or edelfosine treatment of COS7 cells.** Cells were incubated with 50 µM polyfosine, edelfosine or carrier for the indicated times. Mitotracker, whose accumulation depends on mitochondrial activity and vitality, was added prior fixation and fluorescence microscopy (A). Merged color images show green nuclei and red mitochondria, stained by DAPI or Mitotracker, respectively. Bars, 50 µm. (B) Cells with fragmented mitochondria were counted from microscopy images (70–200 cells total for each time point).
(TIF)

Figure S4 **Isolation of mitochondria from COS7 cells incubated with polyfosine or edelfosine.** Cells incubated with carrier (control) or 50 µM polyfosine or edelfosine for 1 h were harvested and homogenized. A crude mitochondria pellet was prepared as described under Material and Methods and separated from a cytosol-enriched supernatant (PMS, post mitochondrial supernatant). The crude mitochondria fraction (crude) was loaded onto a Percoll gradient and centrifuged again (150,000×g; 25 min) before 9 fractions were collected from the top. Aliquots were analyzed by SDS-PAGE and Western blotting for the mitochondrial proteins cytochrome c and cytochrome c oxidase subunit IV (COX IV) and calnexin, an ER marker protein. Note, that in contrast to control cells the mitochondria of polyfosine or edelfosine treated cells cannot be separated from the ER by density gradient centrifugation.
(TIF)

Figure S5 **Disintegrated, blebby mitochondria and condensed nuclei of COS7 cells upon polyfosine treatment.** Cells were incubated with 50 µM polyfosine for 5 h. For the last 15 min before fixation the incubation medium was supplemented with 20 nM Mitotracker dye whose accumulation to mitochondria depends on their activity and vitality. The vitality and morphology of mitochondria was analyzed by fluorescence microscopy. Merged color images show nuclei in green, mitochondria in red, stained by DAPI or Mitotracker, respectively. Bar, 50 µm.
(TIF)

Movie S1 **UV light induced changes of cell morphology upon polyfosine treatment.** COS7 cells were incubated with carrier (upper left) or 50 µM of lyso-ether PC (upper right), edelfosine (lower left) or polyfosine (lower right). Living cells were followed by phase contrast video microscopy for 33 min. During the course of the experiment the cells were constantly illuminated by broadband UV light from a second light source, a Xenon lamp. Bar, 20 µm. Note the fragmentation of mitochondria (lower left and right) and the disintegration of mitochondria and nuclei (lower right).
(MOV)

Text S1 (DOC)
