# Supplementary figures and images for: Exogenous Ether Lipids Predominantly Target Mitochondria
Source: PLoS One. 2012 Feb 14;7(2):e31342. doi: 10.1371/journal.pone.0031342 (PMC3279356; doi:10.1371/journal.pone.0031342)

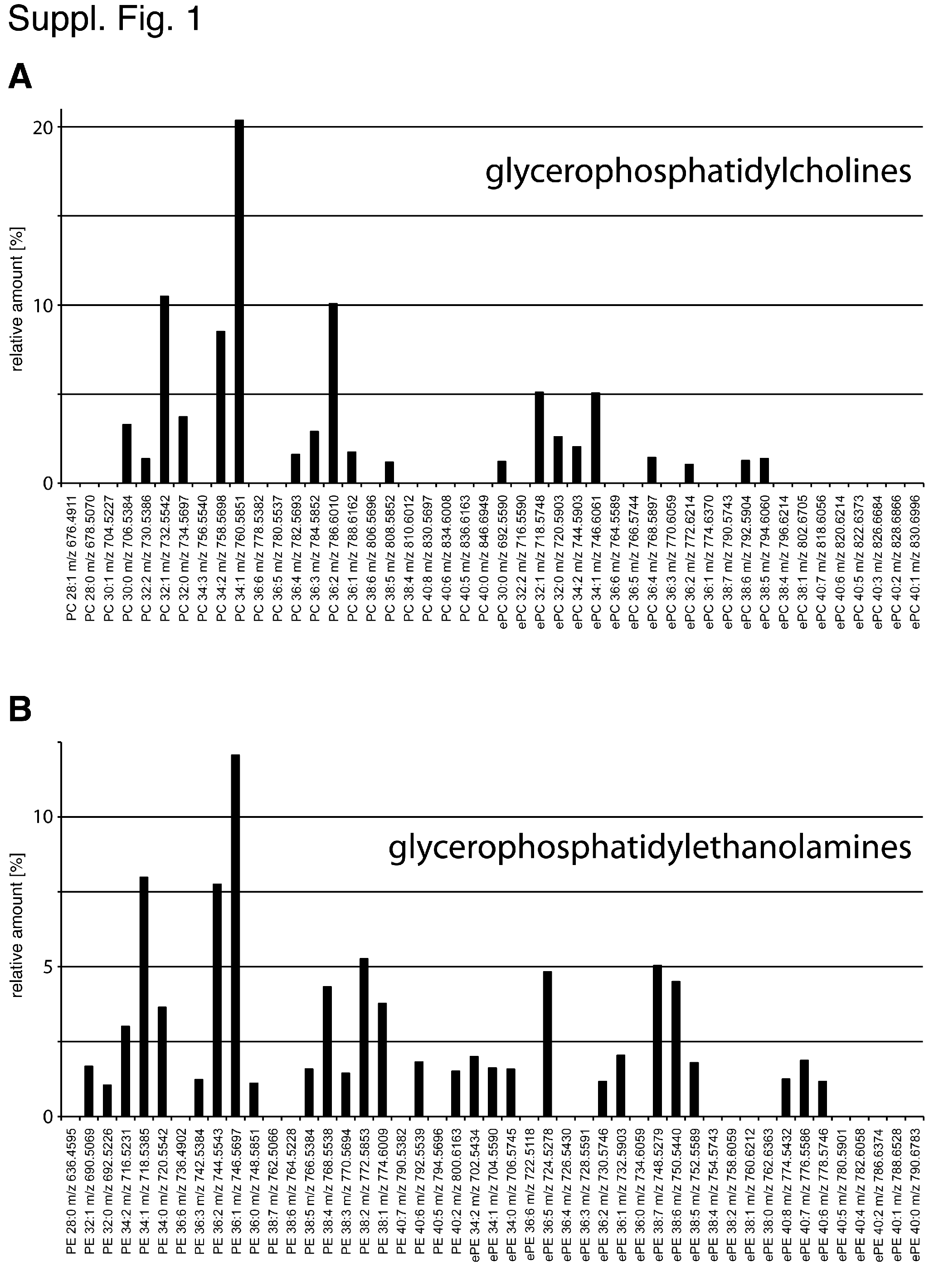

Supplement: Figure S1 — Analysis of glycerophosphatidylcholine and glycerophosphatidylethanolamine lipids species of COS7 cells by MS. Cells were grown in DMEM containing 4.5 g/ml glucose, GlutaMax I, pyruvate and 10% FCS. Total lipid extracts of cells were analyzed for the PC and ePC (A), or PE and ePE (B) species by high-resolution FT MS analysis. The intensity of each species was quantified as a fraction of the sum of all glycerophosphatidylcholine (A) or glycerophosphatidylethanolamine (B) species monitored. Lipid species with a relative amount of less than one percent are omitted. (TIF) [file pone.0031342.s001.tif]

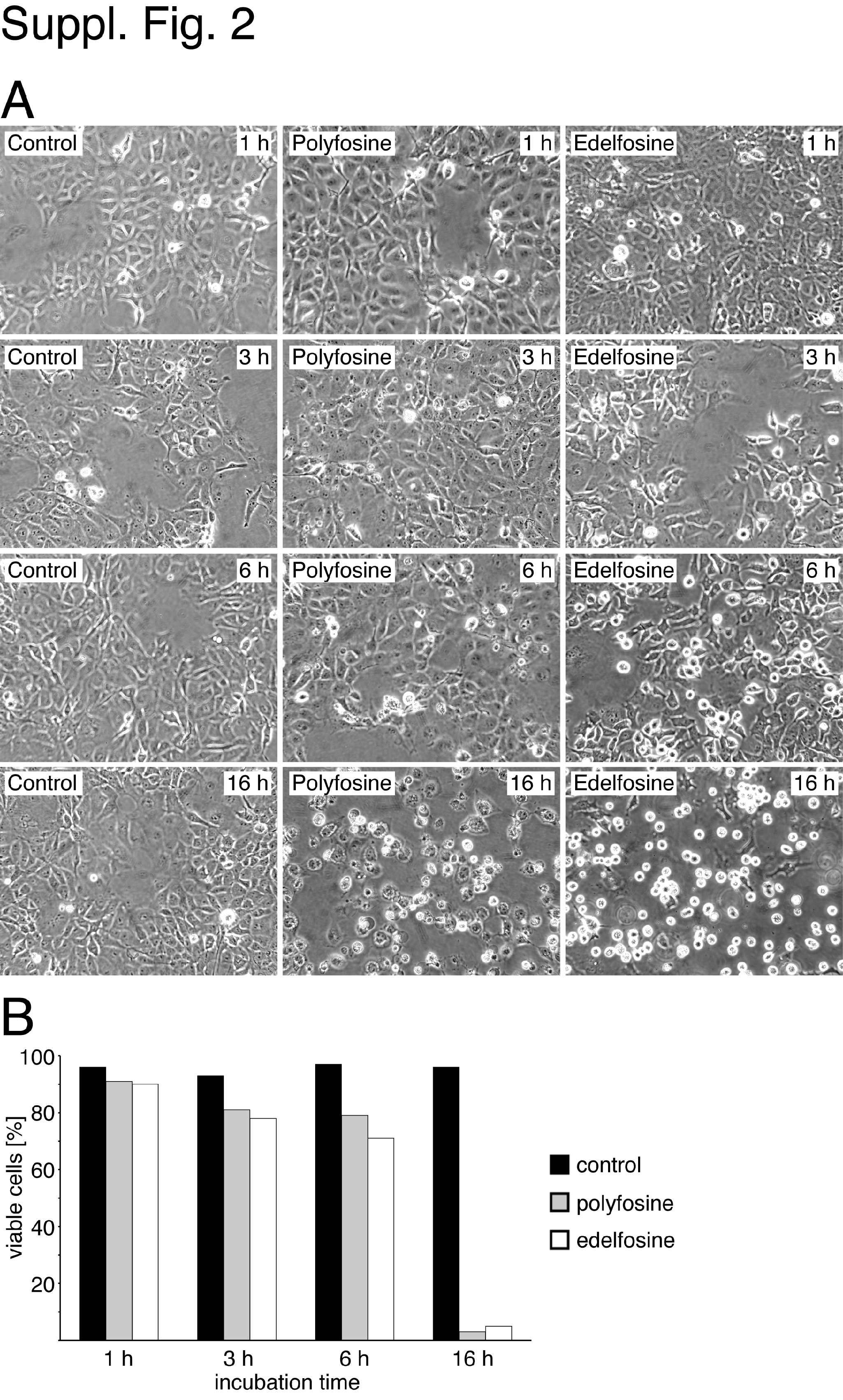

Supplement: Figure S2 — Analysis of the viability of COS7 cells upon treatment with polyfosine or edelfosine. Cells were incubated with 10 µM of polyfosine, edelfosine or carrier as control for indicated times. Representative relief contrast microscopy images of the morphological appearance of the cells are shown (A). Note, that after 3–6 h of incubation with either polyfosine or edelfosine cells were rounding up and detaching from the support presumably by apoptosis-induced detachment. The nuclei appeared granular and fragmented. The number of apoptotic cells as judged by their morphology (detached or rounded up cells with granular fragmented nuclei) were counted and used to calculate the percentage of viable cells (B). (TIF) [file pone.0031342.s002.tif]

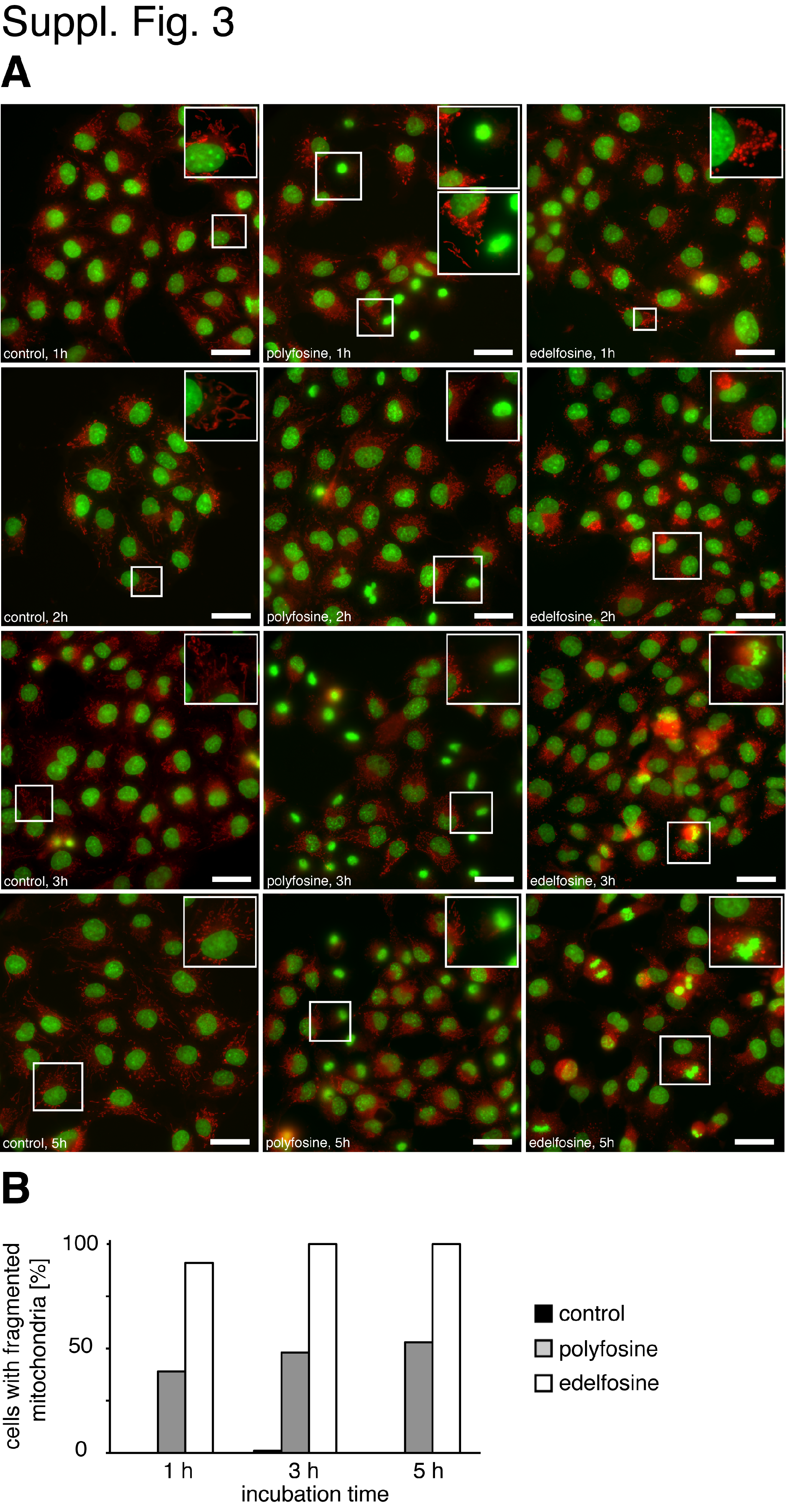

Supplement: Figure S3 — Morphological changes of mitochondria and nuclei upon polyfosine or edelfosine treatment of COS7 cells. Cells were incubated with 50 µM polyfosine, edelfosine or carrier for the indicated times. Mitotracker, whose accumulation depends on mitochondrial activity and vitality, was added prior fixation and fluorescence microscopy (A). Merged color images show green nuclei and red mitochondria, stained by DAPI or Mitotracker, respectively. Bars, 50 µm. (B) Cells with fragmented mitochondria were counted from microscopy images (70–200 cells total for each time point). (TIF) [file pone.0031342.s003.tif]

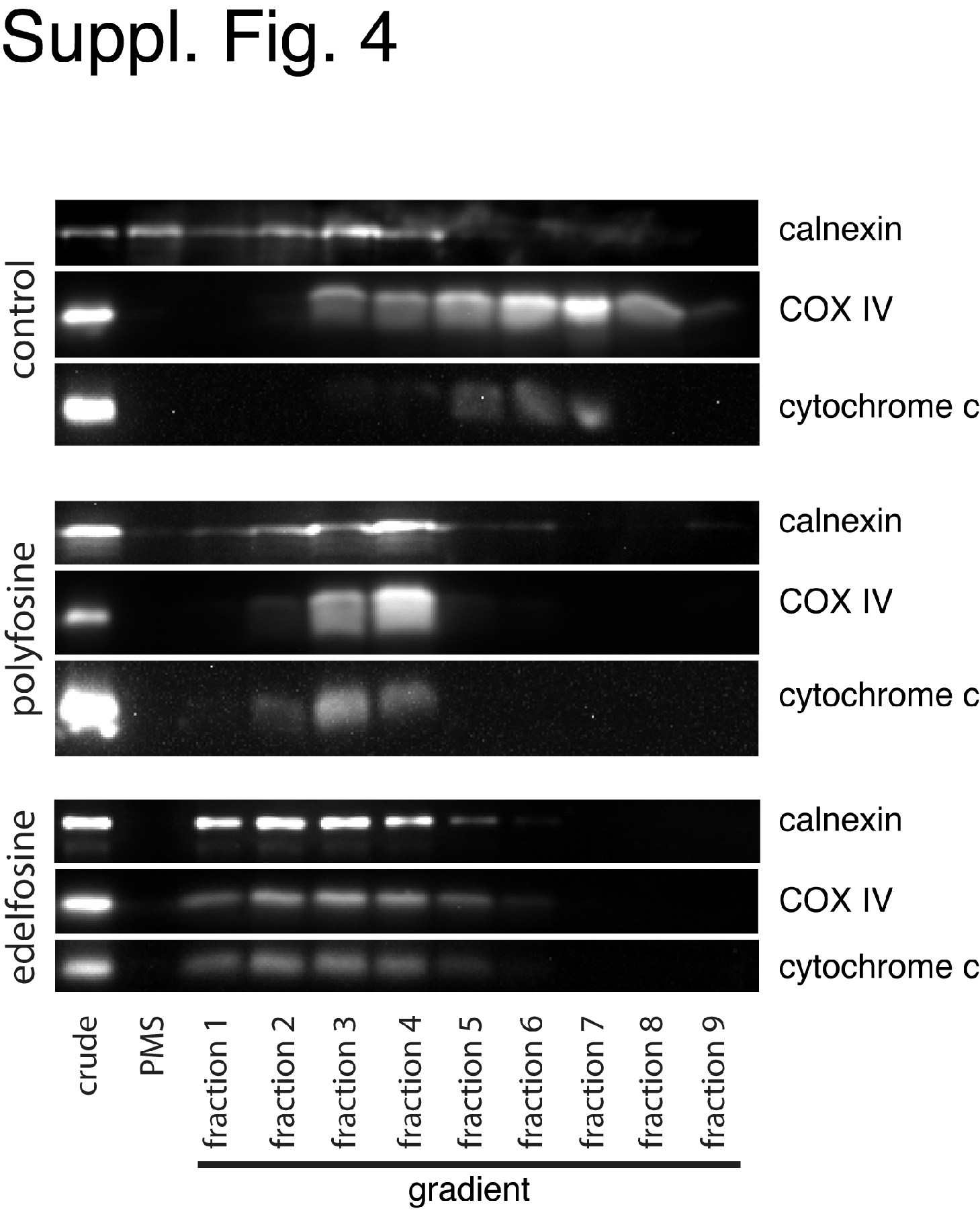

Supplement: Figure S4 — Isolation of mitochondria from COS7 cells incubated with polyfosine or edelfosine. Cells incubated with carrier (control) or 50 µM polyfosine or edelfosine for 1 h were harvested and homogenized. A crude mitochondria pellet was prepared as described under Material and Methods and separated from a cytosol-enriched supernatant (PMS, post mitochondrial supernatant). The crude mitochondria fraction (crude) was loaded onto a Percoll gradient and centrifuged again (150,000×g; 25 min) before 9 fractions were collected from the top. Aliquots were analyzed by SDS-PAGE and Western blotting for the mitochondrial proteins cytochrome c and cytochrome c oxidase subunit IV (COX IV) and calnexin, an ER marker protein. Note, that in contrast to control cells the mitochondria of polyfosine or edelfosine treated cells cannot be separated from the ER by density gradient centrifugation. (TIF) [file pone.0031342.s004.tif]

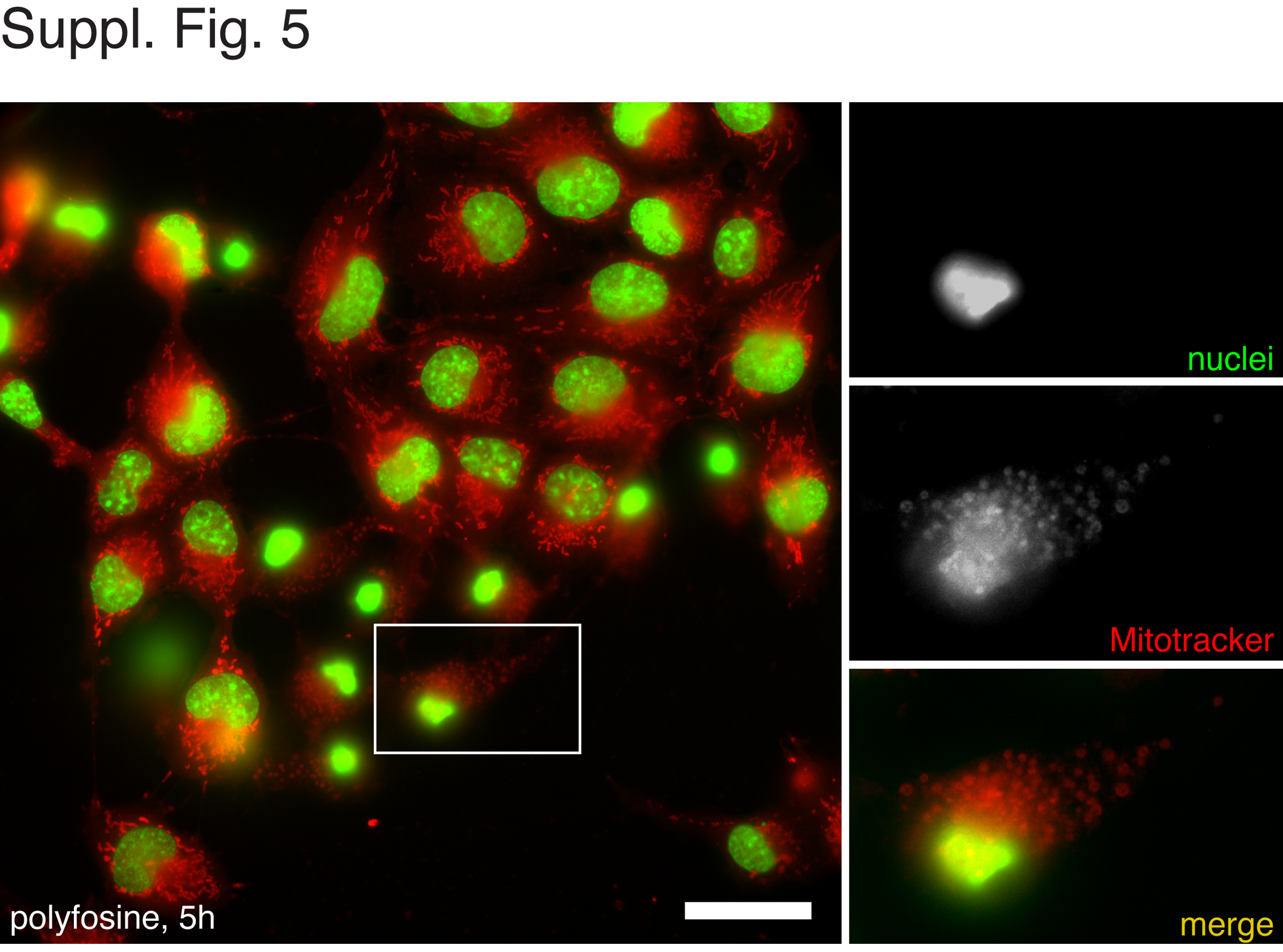

Supplement: Figure S5 — Disintegrated, blebby mitochondria and condensed nuclei of COS7 cells upon polyfosine treatment. Cells were incubated with 50 µM polyfosine for 5 h. For the last 15 min before fixation the incubation medium was supplemented with 20 nM Mitotracker dye whose accumulation to mitochondria depends on their activity and vitality. The vitality and morphology of mitochondria was analyzed by fluorescence microscopy. Merged color images show nuclei in green, mitochondria in red, stained by DAPI or Mitotracker, respectively. Bar, 50 µm. (TIF) [file pone.0031342.s005.tif]
