# Supplementary material for: Exogenous Ether Lipids Predominantly Target Mitochondria
Source: PLoS One. 2012 Feb 14;7(2):e31342. doi: 10.1371/journal.pone.0031342 (PMC3279356; doi:10.1371/journal.pone.0031342)
Supplement: Text S1 — (DOC) [file pone.0031342.s007.doc]

Supplemental text

Hydrocarbon chains of polyenes are denoted as follows: c, indicating the cis configuration of the ω-9 double bond, followed by the number of carbon atoms and the number of double bonds, separated by a colon. All polyenes used in this study are pentaenes featuring conjugated double bonds at ω-9, ω-7, ω-5, ω-3 and ω-1 positions; the latter four in trans configuration.

A. Synthesis of sn1-O-16:5-sn2-lyso-PC and sn1-O-16:5-sn2-lyso-PE

(1) 1-O-(hexadeca-6,8,10,12,14-pentaenyl)-3-O-TBDPS-glycerol (Mr 544). sn-1-O-(hexadeca-6,8,10,12,14-pentaenyl)-glycerol (25) (920 mg, 3 mmol) was dissolved in 20 ml THF. Imidazole (6.1 g, 9 mmol) was added, followed by dropwise addition of 1.24 g (4.5 mmol) tert-butyldiphenylsilylchloride (TBDPS-Cl). After 8h, 1 ml of MeOH was added. After 30 min, 20 ml of hexane were added to precipitate imidazole chloride, followed by purification on silica gel (hexane/THF/ethyl acetate 12/1/1) to obtain 1.11 g (2.0 mmol) of (1).

(2) 1-O-(hexadeca-6,8,10,12,14-pentaenyl)-2-palmitoyl-3-O-TBDPS-glycerol (Mr 782). To a solution of 0.9 g (1.65 mmol) (1) in 5 ml THF and 5 ml DCM, 1 ml of pyridine, 100 mg dimethylaminopyridine and 1 ml palmitoylchloride were added. After 2 h, the solvent was removed and the residue chromatographed on silica gel in hexane/ethyl acetate 98/2 to obtain 750 mg (0.96 mmol) of (2).

(3) 1-O-(hexadeca-6,8,10,12,14-pentaenyl)-2-palmitoyl-glycerol (Mr 544). A solution of 750 mg (0.96 mmol) of (2) in 10 ml THF was stirred with 3 mmol of acetic acid and 3 mmol of TBAF for 6 h at RT. The solvent was removed and the residue chromatographed on silica gel (1^st^ hexane/ethyl acetate 96/4, 2^nd^ 90/10) to give 400 mg (0.74 mmol) of (3).

(4) 1-O-(hexadeca-6,8,10,12,14-pentaenyl)-2-palmitoyl-PC (Mr 710). 400 mg (0.74 mmol) of (3) were dissolved in 1 ml THF. This solution was added to a solution of 0.1 ml (1.1 mmol) of POCl3 and 0.12 ml (1.5 mmol) pyridine in 2 ml DCM for 30 min, followed by addition of 1 g (4 mmol) of choline tosylate and 1 ml of pyridine. After stirring for 16 h, the solvent was removed and the product isolated by chromatography on silica gel (CHCl_3_/MeOH/THF/H_2_O 65/25/10/4) to give 70 mg (0.1 mmol) of pure (4). ES-MS positive ion mode M/Z 710.5 [M+H]^+^, MS/MS 184.07 (phosphorylcholine^+^); negative ion mode 744.47 [M+Cl]^-^, MS/MS 255.2 (palmitate^-^). UV: 346.2 TLC (silica gel, CHCl_3_/MeOH/THF/H_2_O 65/25/10/4): co-migrates with DOPC

(5) 1-O-(hexadeca-6,8,10,12,14-pentaenyl)-2-lyso-PC (Mr 472). 14 mg (0.02 mmol) of (4) were dissolved in 2 ml of MeOH/THF 1/1 and treated with 0.06 ml 1 M KOH for 3 h at 38 degrees C. The product was isolated by chromatography on silica gel (1^st^ CHCl_3_/THF/MeOH/H_2_O 50/15/25/3, 2^nd^ 40/10/40/10) to give 8.5 mg (5). ES-MS positive ion mode M/Z 472.28 [M+H]^+^; MS/MS 184.07 (phosphorylcholine^-^), UV: 346.3 nm. TLC (silica gel, CHCl_3_/MeOH/THF/H_2_O 45/35/15/8): Rf 0.27, co-migrates with 1-oleoyl-2-lyso-PC.

(6) 1-O-(hexadeca-6,8,10,12,14-pentaenyl)-2-lyso-PE (Mr 430). (6) was synthesized from (3) using the procedure by Eibl (26), followed by alkaline cleavage as above to give (6). ES-MS positive ion mode M/Z 430.24 [M+H]^+^; negative ion mode 428.24 [M-H]^-^, MS/MS 140.02 (phosphorylethanolamine^-^), no signals of fatty acids.

B. Synthesis of polyfosine, 1-O-(hexadeca-6,8,10,12,14-pentaenyl)-2-O-methyl-PC

(7) 1-O-(hexadeca-6,8,10,12,14-pentaenyl)-2-O-methyl-glycerol (Mr 320). To a solution of 1.6 g (3 mmol) of (1) in 40 ml THF, 10 ml of 1M lithium hexamethyldisilazane and 1 ml CH_3_I were added. After 16 h, another 5 ml 1M lithium hexamethyldisilazane and 5 ml of CH_3_I were added, the mixture warmed to 40 degrees C and stirred for 3 h. The solvent was evaporated and the product isolated by silica gel chromatography (Hexane/THF 10/1) to yield 1.1 g (2 mmol) of 1-O-(hexadeca-6,8,10,12,14-pentaenyl)-2-O-methyl-3-O-TBDPS-glycerol.

The protective group was removed as above by treatment with acetic acid and TBAF to obtain 510 mg (1,6 mmol) (7).

(8) 1-O-(hexadeca-6,8,10,12,14-pentaenyl)-2-O-methyl-PC (polyfosine, Mr 486). A solution of 500 mg (1.6 mmol) (7) and 0.17 ml pyridine in 5 ml THF was added to a solution of 0.2 ml (2.2 mmol) POCl_3_ in 5 ml DCM. After 30 min stirring at 45 degrees C, 1 ml pyridine and 1 g (4 mmol) choline tosylate were added. After stirring for 24 h at RT, the solvents were removed and the product purified by silica gel chromatograph (gradient of CHCl_3_/MeOH/THF/H_2_O 50/25/15/4 to 40/40/10/8) to obtain 64 mg of pure (8). ES-MS positive ion mode M/Z 486.3 [M+H]^+^, MS/MS 184.07 (phosphorylcholine^+^). UV: 346.1 nm. TLC (silica gel, CHCl_3_/MeOH/THF/H_2_O 45/35/15/8): Rf 0.36

**Supplemental material legends**

Supplemental Figure 1. Analysis of glycerophosphatidylcholine and glycerophosphatidylethanolamine lipids species of COS7 cells by MS. Cells were grown in DMEM containing 4.5 g/ml glucose, GlutaMax I, pyruvate and 10% FCS. Total lipid extracts of cells were analyzed for the PC and ePC (A), or PE and ePE (B) species by high-resolution FT MS analysis. The intensity of each species was quantified as a fraction of the sum of all glycerophosphatidylcholine (A) or glycerophosphatidylethanolamine (B) species monitored. Lipid species with a relative amount of less than one percent are omitted.

Supplemental Figure 2. Analysis of the viability of COS7 cells upon treatment with polyfosine or edelfosine. Cells were incubated with 10 μM of polyfosine, edelfosine or carrier as control for indicated times. Representative relief contrast microscopy images of the morphological appearance of the cells are shown (A). Note, that after 3-6 h of incubation with either polyfosine or edelfosine cells were rounding up and detaching from the support presumably by apoptosis-induced detachment. The nuclei appeared granular and fragmented. The number of apoptotic cells as judged by their morphology (detached or rounded up cells with granular fragmented nuclei) were counted and used to calculate the percentage of viable cells (B).

Supplemental Figure 3. Morphological changes of mitochondria and nuclei upon polyfosine or edelfosine treatment of COS7 cells. Cells were incubated with 50 μM polyfosine, edelfosine or carrier for the indicated times. Mitotracker, whose accumulation depends on mitochondrial activity and vitality, was added prior fixation and fluorescence microscopy (A). Merged color images show green nuclei and red mitochondria, stained by DAPI or Mitotracker, respectively. Bars, 50 μm. (B) Cells with fragmented mitochondria were counted from microscopy images (70-200 cells total for each time point).

Supplemental Figure 4. Isolation of mitochondria from COS7 cells incubated with polyfosine or edelfosine. Cells incubated with carrier (control) or 50 μM polyfosine or edelfosine for 1 h were harvested and homogenized. A crude mitochondria pellet was prepared as described under Material and Methods and separated from a cytosol-enriched supernatant (PMS, post mitochondrial supernatant). The crude mitochondria fraction (crude) was loaded onto a Percoll gradient and centrifuged again (150,000xg; 25 min) before 9 fractions were collected from the top. Aliquots were analyzed by SDS-PAGE and Western blotting for the mitochondrial proteins cytochrome c and cytochrome c oxidase subunit IV (COX IV) and calnexin, an ER marker protein. Note, that in contrast to control cells the mitochondria of polyfosine or edelfosine treated cells cannot be separated from the ER by density gradient centrifugation.

Supplemental Figure 5. Disintegrated, blebby mitochondria and condensed nuclei of COS7 cells upon polyfosine treatment. Cells were incubated with 50 μM polyfosine for 5 h. For the last 15 min before fixation the incubation medium was supplemented with 20 nM Mitotracker dye whose accumulation to mitochondria depends on their activity and vitality. The vitality and morphology of mitochondria was analyzed by fluorescence microscopy. Merged color images show nuclei in green, mitochondria in red, stained by DAPI or Mitotracker, respectively. Bar, 50 μm.

Supplemental Movie 1. UV light induced changes of cell morphology upon polyfosine treatment. COS7 cells were incubated with carrier (upper left) or 50 μM of lyso-ether PC (upper right), edelfosine (lower left) or polyfosine (lower right). Living cells were followed by phase contrast video microscopy for 33 min. During the course of the experiment the cells were constantly illuminated by broadband UV light from a second light source, a Xenon lamp. Bar, 20 μm. Note the fragmentation of mitochondria (lower left and right) and the disintegration of mitochondria and nuclei (lower right).
